# *In EBUS Signo Vinces:* New Indications in Thoracic Oncology for Mediastinal Lymph Node Staging Using Endobronchial Ultrasound

**DOI:** 10.3389/fonc.2022.934986

**Published:** 2022-07-08

**Authors:** Juliana Guarize, Lorenzo Spaggiari, Luca Bertolaccini

**Affiliations:** ^1^ Unit of Interventional Pneumology, IEO, European Institute of Oncology IRCCS, Milan, Italy; ^2^ Department of Thoracic Surgery, IEO, European Institute of Oncology IRCCS, Milan, Italy; ^3^ Department of Oncology and Hemato-Oncology, University of Milan, Milan, Italy

**Keywords:** endo-bronchial ultrasound, lung cancer, lung cancer staging, tailored therapy, bronchoscopy

## Introduction

Lung cancer continues to be the leading cause of cancer-related death globally. Quick access to tailored diagnosis could expedite the molecular investigation process and improve overall results (1). A standardised strategy for staging and diagnosis is a fundamental component of lung cancer management. However, there is little doubt that molecular tissue diagnosis approaches are critical for many patients’ outcomes (2, 3).

Over the last decade, the importance of endobronchial ultrasound (EBUS) in lung cancer management pathways has evolved in lockstep with significant breakthroughs in molecular profiling and available treatments. Multiple molecular tests are now required to guide the treatment plan. Additionally, immunological testing and research are advancing rapidly, and targeted medicines and immunotherapies are being investigated more frequently to treat early-stage lung cancer. EBUS transbronchial needle aspirations (TBNAs) are increasingly used to diagnose and obtain tissue-based biomarkers. There should be a tight collaboration between the pulmonologist, surgeon, medical oncologist, and pathologist. Ideally, the pathologist can simultaneously send samples for diagnosis, staging, and molecular analysis. These results are available to the medical oncologist during the initial appointment rather than being ordered *post hoc*, further delaying therapeutic decision-making. Therefore, there is an increasing demand for high-quality EBUS-TBNA to meet these objectives, many of which are not addressed by current EBUS recommendations (4).

*In hoc signo vinces* (Latin for *in this sign you will win*) is a phrase with which the Greek motto *τούτῳ νίκα* is commonly translated as *win with this*. The phrase appeared in Constantine’s dream, together with a flaming cross, shortly before he moved from Gaul to Rome to wage war against Maxentius (immediately following the dream, he created the banner with the monogram of Christ drawn on it). Constantine had the vision in a dream on the eve of the decisive Battle of the Milvian Bridge (5). In this Opinion Paper for the *Frontiers in Oncology* journal, we will describe our vision of the new flaming approach of EBUS to lung cancer staging and management.

## New indications of endobronchial ultrasound in thoracic oncology

Molecular analysis performed on EBUS-TBNA specimens was comparable to that performed on surgical specimens regarding sensitivity or diagnostic accuracy. Due to the low invasiveness and feasibility of repeated procedures, EBUS-TBNA is ideal for establishing a cell-type diagnosis and providing adequate specimens for molecular assay in locally advanced lung cancer patients. The procedure’s accuracy and minimal risk of complications make it suitable for low-performance-status patients who could still benefit from targeted therapy (6).

**Figure 1:**
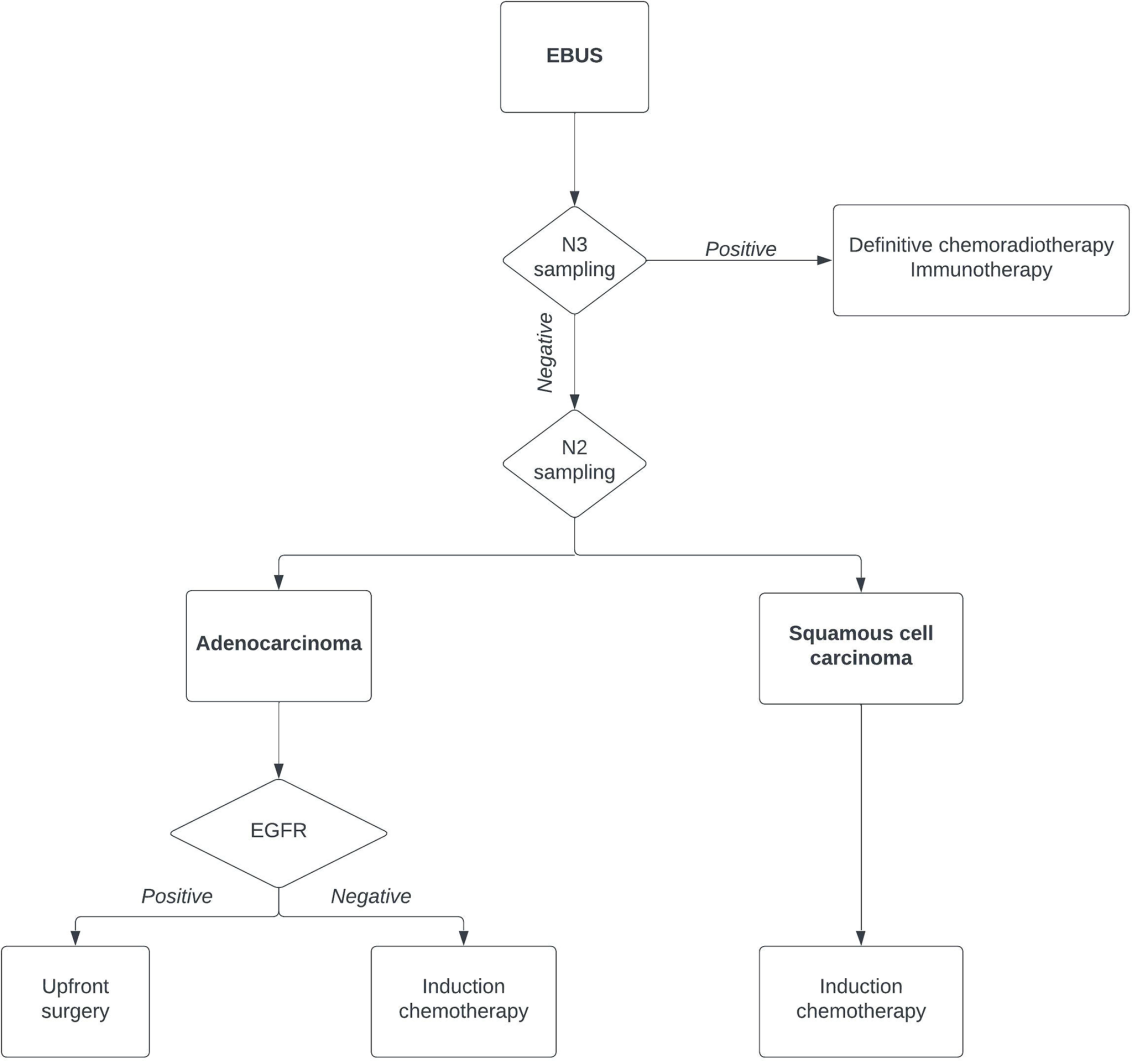
Flowchart of lung cancer diagnosis using endobronchial ultrasound. EGFR, epidermal growth factor receptor.

Therefore, the current role of EBUS is not only as a diagnostic tool to confirm the pathological N2 station (**Figure 1**). The current approach should exclude a pathological N3 involvement, and the patient could undergo definitive chemoradiotherapy and/or immunotherapy. If an N3 contralateral involvement is excluded, the role of EBUS in N2 lymph nodes is a tailored diagnostic tool. After the EBUS-TBNA diagnosis, a patient with squamous cell carcinoma should undergo induction therapy. On the other hand, an epidermal growth factor receptor (EGFR)-positive patient should undergo upfront surgery with a subsequent tyrosine kinase inhibitor treatment with great results (7, 8).

A likelihood ratio should also be calculated to assess the potential utility of the EBUS-TBNA and how likely it is that a patient has pathological lymph nodes. The likelihood ratio is the ratio of the probability that a test result is correct to the probability that the test result is incorrect. The sensitivity and specificity of the test are the values used to generate a likelihood ratio, which is calculated for both positive and negative test results. The advantage of the likelihood ratio with four categories over a binary test is the clinical interpretation and usability of the data (9). EBUS-TBNA sensitivity and diagnostic accuracy in locally advanced lung cancers were 98.5% when pathological diagnosis and mutational status were considered. Additionally, EBUS-TBNA produced adequate material for molecular analysis in 98% of instances with advanced-stage adenocarcinomas (9).

## Discussion

Blinded quantitative analysis of tumour size as a predictor of lymph nodes metastasis in T1 lung tumours discovered that even among tiny tumours, a high prevalence of N2 or N3 metastasis persists, and there is no clear definition of central lesion that mitigates this risk (10).

Accurate mediastinal lung cancer staging is critical since it directly affects prognosis and treatment options. Multimodal staging of preoperative mediastinal lymph nodes in lung cancer is critical for accurate staging and selecting the most appropriate treatment. The diagnostic usefulness of 2-[fluorine-18]fluoro-2-deoxy-D-glucose PET (^18^F-FDG PET) is superior to that of the chest CT scan. In preoperative mediastinal lymph nodes, the negative predictive value of ^18^F-FDG PET/CT is reliable and comparable to the negative predictive value of EBUS-TBNA. The maximal standardised uptake value (SUVmax) of mediastinal lymph nodes can aid in the prediction of metastases; however, positive ^18^F-FDG PET/CT mediastinal lymph nodes should be confirmed histopathologically, particularly if such a result would alter the treatment strategy (11). EBUS-TBNA has been better than any commonly used procedure for mediastinal lung cancer staging (12). The primary disadvantages of EBUS and TBNA are that certain lymph node stations, such as levels 5 and 6, are inaccessible by either technique. Mediastinoscopy is not an appropriate alternative to EBUS-TBNA in centres where it is not accessible. These hospitals should refer patients to high-volume centres with a significant experience in the procedure (13).

EBUS-TBNA sampling of tumours is commonly performed with numerous samples taken from multiple sites, allowing for a more considerable breadth of assessment and overcoming intra- and inter-lesional heterogeneity. EBUS-guided bronchoscopy is a minimally invasive procedure that allows repeated tumour tissue collection. When paired with the rapid-on-site examination (ROSE), EBUS-guided sampling can rapidly diagnose cancer with high sensitivity and specificity. Radial probe EBUS is used to sample pulmonary parenchymal lesions, a unique technique that incorporates both cytologic and histologic sampling. Additionally, EBUS-TBNA sampling has been used to characterise the immunophenotypic properties of tumour-draining lymph nodes in not-small cell lung cancer (14).

In published studies, the diagnostic accuracy of EBUS-TBNA for mediastinal and hilar lymph node pathology was 81.2%. Cancer, lung cancer, and benign lesions were diagnosed with disease-specific accuracy rates of 81.7%, 84.3%, and 78.9%, respectively. The sensitivities for all diseases, cancer, lung cancer, and benign were, respectively, 55.1%, 48.8%, 48.3%, and 60%. The negative predictive values for all diseases, cancer, lung cancer, and benign were 75.7, 77.2, 81.2, and 69.2%, respectively. The most crucial use of EBUS-TBNA is diagnosing lymph node metastases in lung cancer; the sensitivity of EBUS-TBNA in this regard ranges from 46% to 96% (15).

In an ungraded consensus-based statement, CHEST guidelines issued in 2016 stated that this approach is suitable for obtaining a differential diagnosis between lymphoid and epithelial neoplasms in cases of suspected lymphoma (16). The American College of Chest Physicians (16), the European Society for Medical Oncology (17), the European Society of Thoracic Surgeons (18), and the National Comprehensive Cancer Network (19) currently recommend EBUS-TBNA as the method of choice for invasive mediastinal staging, even though mediastinoscopy was once regarded as the optimal method.

Recently, immunotherapy with monoclonal antibodies directed against the programmed death-ligand 1 (PD-L1) has become a promising treatment option in patients with advanced non-small cell lung cancer in terms of overall survival. This is critical for identifying patients who will benefit most from immunotherapy. In recent years, minimally invasive treatments have become the gold standard for diagnosing and staging lung cancer patients. Nevertheless, EBUS TBNA procedures frequently offer the necessary information, from tissue samples to molecular analysis, with few complications. EBUS-TBNA cytologic specimens have been effectively used to assess a variety of molecular targets, including EGFR, Anaplastic Lymphoma Kinase (ALK), and c-ros oncogene 1 (ROS-1). They are acceptable and equivalent to histologic samples for assessing target markers (20).

While EBUS-TBNA has been established and widely implemented in clinical practice, high-quality research is still required. Randomised controlled prospective studies are critical for defining the benefits of technological developments such as navigational approaches, new robotic platforms, needle designs, and cytopathological processing modifications. The design of future studies should reflect the growing demands of lung cancer precision diagnostics by shifting the significant focus away from diagnostic yield and cellular quality and quantity (21). Implementation research will improve procedural procedures and the broader adoption of best practices (4).

## Conclusions

EBUS-TBNA is not only a technology but also a way of thinking about lung cancer management. The feasibility and ideal results of EBUS-TBNA depend highly on the cytopathology and pulmonologist’s expertise and how the technique is performed. Patient sedation and ROSE are critical and should always be used. With experienced hands and multidisciplinary experience, EBUS-TBNA provides all the necessary information that a medical oncologist requires for optimal medical treatment without requiring additional invasive procedures (6). EBUS specimens can be obtained from a single procedure for pathological diagnosis, staging, and comprehensive molecular assessment, thereby establishing the foundation for the personalised therapy era, which combines minimally invasive procedures with biological agents to achieve the best oncological outcomes. EBUS-TBNA represents the best diagnostic approach in many different clinical scenarios in high-volume thoracic oncology centres and is valid and accurate in underpinning modern oncological therapy, guiding the best and tailored treatment options in lung cancer (20).

## Author Contributions

All authors listed have made a substantial, direct, and intellectual contribution to the work, and approved it for publication.

## Funding

This work was partially supported by the Italian Ministry of Health with *Ricerca Corrente* and *5x1000* funds.

## Conflict of Interest

The authors declare that the research was conducted in the absence of any commercial or financial relationships that could be construed as a potential conflict of interest.

## Publisher’s Note

All claims expressed in this article are solely those of the authors and do not necessarily represent those of their affiliated organizations, or those of the publisher, the editors and the reviewers. Any product that may be evaluated in this article, or claim that may be made by its manufacturer, is not guaranteed or endorsed by the publisher.

## References

[1] Bertolaccini L Ciani O Prisciandaro E Sedda G Spaggiari L. Lung Cancer Stage Distribution From Before Covid-19 Through 18 Months of the Pandemic: The Experience of a Large-Volume Oncological Referral Centre. Eur J Surg Oncol (2022) 48(2):470–1. doi: 10.1016/j.ejso.2021.09.024 PMC8487321 34627642

[2] King J Shah D Hewitt K Punjabi A Marshall K Balata H. The Diagnostic Pathway in Lung Cancer Patients With Best Supportive Care Decisions: Are There Lessons to Be Learnt? Clin Med (Lond) (2022) 22(3):246–50. doi: 10.7861/clinmed.2021-0160 PMC9135074 35443968

[3] Ciani O Federici CB. Value Lies in the Eye of the Patients: The Why, What, and How of Patient-Reported Outcomes Measures. Clin Ther (2020) 42(1):25–33. doi: 10.1016/j.clinthera.2019.11.016 31932079

[4] Oezkan F Eisenmann S Darwiche K Gassa A Carbone DP Merritt RE. Linear Endobronchial Ultrasound in the Era of Personalized Lung Cancer Diagnostics-A Technical Review. J Clin Med (2021) 10(23). doi: 10.3390/jcm10235646 PMC8658311 34884348

[5] In Hoc Signo Vinces in Vocabolario - Treccani: @Treccani (2022). Available at: https://www.treccani.it/vocabolario/in-hoc-signo-vinces.

[6] Guarize J Casiraghi M Donghi S Diotti C Vanoni N Romano R. Endobronchial Ultrasound Transbronchial Needle Aspiration in Thoracic Diseases: Much More Than Mediastinal Staging. Can Respir J (2018) 2018:4269798. doi: 10.1155/2018/4269798 29686741 PMC5857308

[7] Wu YL Tsuboi M He J John T Grohe C Majem M. Osimertinib in Resected Egfr-Mutated Non-Small-Cell Lung Cancer. N Engl J Med (2020) 383(18):1711–23. doi: 10.1056/NEJMoa2027071 32955177

[8] Bertolaccini L Prisciandaro E Sedda G Girelli L Spaggiari L. 89p Long-Term Clinical Outcomes and Prognostic Factors of Upfront Surgery as a First-Line Therapy in Pathological N2 Nsclc. J Thorac Oncol (2021) 16(4):S744. doi: 10.1016/s1556-0864(21)01931-6 PMC9366141 35965495

[9] Ost DE. Interpretation and Application of the Likelihood Ratio to Clinical Practice in Thoracic Oncology. J Bronchol Interv Pulmonol (2022) 29(1):62–70. doi: 10.1097/LBR.0000000000000788 34162800

[10] DuComb EA Tonelli BA Tuo Y Cole BF Mori V Bates JHT. Evidence for Expanding Invasive Mediastinal Staging for Peripheral T1 Lung Tumors. Chest (2020) 158(5):2192–9. doi: 10.1016/j.chest.2020.05.607 PMC8173766 32599066

[11] Al-Ibraheem A Hirmas N Fanti S Paez D Abuhijla F Al-Rimawi D. Impact of (18)F-Fdg Pet/Ct, Ct and Ebus/Tbna on Preoperative Mediastinal Nodal Staging of Nsclc. BMC Med Imaging (2021) 21(1):49. doi: 10.1186/s12880-021-00580-w 33731050 PMC7967993

[12] Marcoux M Ost DE. What's New in Endobronchial Ultrasound for Mediastinal Staging? Curr Opin Pulm Med (2020) 26(4):346–58. doi: 10.1097/MCP.0000000000000686 32487873

[13] Jiang L Huang W Liu J Harris K Yarmus L Shao W. Endosonography With Lymph Node Sampling for Restaging the Mediastinum in Lung Cancer: A Systematic Review and Pooled Data Analysis. J Thorac Cardiovasc Surg (2020) 159(3):1099–108.e5. doi: 10.1016/j.jtcvs.2019.07.095 31590952

[14] Bozinovski S Vannitamby A Rangamuwa K Aujla S Wang H Aloe C. Integrating Endobronchial Ultrasound Bronchoscopy With Molecular Testing of Immunotherapy Biomarkers in Non-Small Cell Lung Cancer. Transl Lung Cancer Res (2021) 10(6):2779–87. doi: 10.21037/tlcr-20-781 PMC8264344 34295677

[15] Murthi M Donna E Arias S Villamizar NR Nguyen DM Holt GE. Diagnostic Accuracy of Endobronchial Ultrasound-Guided Transbronchial Needle Aspiration (Ebus-Tbna) in Real Life. Front Med (Lausanne) (2020) 7:118. doi: 10.3389/fmed.2020.00118 32318581 PMC7154097

[16] Wahidi MM Herth F Yasufuku K Shepherd RW Yarmus L Chawla M. Technical Aspects of Endobronchial Ultrasound-Guided Transbronchial Needle Aspiration: Chest Guideline and Expert Panel Report. Chest (2016) 149(3):816–35. doi: 10.1378/chest.15-1216 26402427

[17] Postmus PE Kerr KM Oudkerk M Senan S Waller DA Vansteenkiste J. Early and Locally Advanced Non-Small-Cell Lung Cancer (Nsclc): Esmo Clinical Practice Guidelines for Diagnosis, Treatment and Follow-Up. Ann Oncol (2017) 28(suppl_4):iv1–iv21. doi: 10.1093/annonc/mdx222 28881918

[18] De Leyn P Dooms C Kuzdzal J Lardinois D Passlick B Rami-Porta R. Revised Ests Guidelines for Preoperative Mediastinal Lymph Node Staging for Non-Small-Cell Lung Cancer. Eur J Cardiothorac Surg (2014) 45(5):787–98. doi: 10.1093/ejcts/ezu028 24578407

[19] Ettinger DS Wood DE Aisner DL Akerley W Bauman JR Bharat A. Non-Small Cell Lung Cancer, Version 3.2022, Nccn Clinical Practice Guidelines in Oncology. J Natl Compr Canc Netw (2022) 20(5):497–530. doi: 10.6004/jnccn.2022.0025 35545176

[20] Guarize J Rocco EG Marinis F Sedda G Bertolaccini L Donghi SM. Prospective Evaluation of Ebus-Tbna Specimens for Programmed Death-Ligand 1 Expression in Non-Small Cell Lung Cancer Patients: A Pilot Study. J Bras Pneumol (2021) 47(4):e20200584. doi: 10.36416/1806-3756/e20200584 34259745 PMC8332653

[21] Bertolaccini L Ciani O Spaggiari L. Comment on the Unbearable Lightness of Difference Between Statistical and Clinical Significance. Ann Surg Open (2022) 3(1):e114. doi: 10.1097/as9.0000000000000122 PMC10431439 37600085

